# Supplementary material for: Chromothripsis during telomere crisis is independent of NHEJ, and consistent with a replicative origin
Source: Genome Res. 2019 May;29(5):737–49. doi: 10.1101/gr.240705.118 (PMC6499312; doi:10.1101/gr.240705.118)
Supplement: Supplemental Material [file supp_gr.240705.118_Supplemental_file_1.zip › contigs/annotated_contigs/DB47/contig.2.DB47_length_635_mean_cov_10.3118110236.docx]

**DB47_length_635_mean_cov_10.3118110236**

```
GGTTCCCCTCCATGTGGACTCCCCTCTTCCTGGGCCAGTGAACGAGGCGAGGCTTCCTCACAGCACTGTGGTCTCAGCATTCCAAGAGT
 >chr19:57560813-57561115 - E=3e-165
GTGGAAAGGAAAGCTGCAAGGCTTCATGGGCCTCACGGCGGCAGGCACATGCTATCGTTTCTACCACGTTCCGTTGCAAAAAAAAAAGT

TGCAGAGCCAGGCCAGAGCCAGGAAACATGGGAGACAGACACCCTTCTACCCTTCTGGAGCATCAAGTGATTTTTGGCCATTTTTAATC

TTCAACATTACATATATATATATATATATAT|ATA|CAAGTTGTTGCAAAAGACATCATTTTGTTTCTTTTTATGGCTGAGTAGTATTC
                                >chr19:57559602-57559939 - E=4e-191
CATGGTGTGTCTATACCACATTTTCCTTATCCACTCTTTGGTCAATGGGTACTTAGGTTGGTTCCCTGTCTTTGCAATTGTGAATTGTG

CTGCTATAAACATATATGTGCAAGTGTCTTTTTCATGTAATGACTTCTTTTCCTTTGTGTAGATACCCAGTAGTGGGATTGCTGGACAG

AATGGAAGTCCTGCTTTTAGTTCTTTAGGGAATCTCTATACTGTTTTCCATAGTGTCAACCAATCATTTTTGAGGGTGCCCCACACTGC

TCAGGTGGTTGTGC
```
